# Real-world safety and efficacy of Anti-VEGF treatment in Brazil

**DOI:** 10.5935/0004-2749.2024-0277

**Published:** 2025-04-07

**Authors:** Mario Cesar Bulla, Daniel Lavinsky

**Affiliations:** 1 Clínica Bulla, São Leopoldo, RS, Brazil; 2 Departamento de Oftalmologia, Faculdade de Medicina, Universidade Federal do Rio Grande do Sul, Porto Alegre, RS, Brazil

**Keywords:** Antiangiogenic drugs, Macular edema, Age-related macular degeneration, Retinal vein occlusion, Patient safety

## Abstract

**Purpose:**

This retrospective study evaluated the safety and efficacy of real-world antiangiogenic therapy for ocular conditions in the private healthcare sector in southern Brazil.

**Methods:**

Medical records from patients who underwent intravitreal anti-vascular endothelial growth factor injections over the past 12 years were reviewed retrospectively. Data collection included the primary diagnoses, drugs administered, injection techniques, adverse effects, and treatment efficacy. Efficacy was assessed by comparing preand posttreatment visual acuity and central subfield thickness in eyes with followup exceeding 2 years.

**Results:**

A total of 1,024 patients, 1,310 treated eyes, and 11,377 injections were analyzed. The injections included aflibercept (6,833), ranibizumab (3,692), bevacizumab (843), and brolucizumab (9), administered either bilaterally (3,696) or unilaterally (7,681). The most common diagnoses were diabetic macular edema, exudative age-related macular degeneration, retinal vein occlusion related macular edema, and proliferative diabetic retinopathy. No endophthalmitis cases were reported. Vitritis with transient visual acuity loss occurred in two cases following aflibercept injections. One retinal detachment case was successfully treated with vitrectomy. The median number of injections per patient was 6 (IQR [interquartile range], 3-13). Among 445 eyes from 328 patients with followup over 2 years (median, 4.05 years; IQR, 2.89-6.29), there was a significant improvement in best-corrected visual acuity from 0.3 to 0.4 (Snellen) (p<0.001) and a reduction in central subfield thickness from 361 to 267 microns (p<0.001). CST comparisons included patients with age-related macular degeneration, diabetic macular edema, and retinal vein occlusion related macular edema.

**Conclusion:**

This real-world study, the largest of its kind in Brazil, confirms the safety and efficacy of antiangiogenic therapies in the southern Brazilian private healthcare system. The findings highlight a low incidence of severe adverse events and outcomes consistent with global studies, supporting the ongoing use of antiangiogenic agents as effective and well-tolerated treatments for various ocular conditions in developing countries.

## INTRODUCTION

Retinal conditions, including age-related macular degeneration (AMD), diabetic retinopathy (DR), and retinal vein occlusion (RVO), represent significant global challenges to vision health^(1)^. These diseases are characterized by abnormal blood vessel growth in the retina, known as angiogenesis, along with increased vascular permeability, which leads to retinal edema and damage. Intravitreal injections of antiangiogenic agents have become the primary treatment for these conditions^(2)^. These drugs work by inhibiting vascular endothelial growth factor (VEGF), a protein that promotes blood vessel formation and increases vascular permeability. By blocking VEGF, these treatments help prevent pathological angiogenesis and its associated effects.

Despite the proven efficacy in randomized clinical trials^(3)^, extensive real-world studies have mostly been conducted at international centers, reporting endophthalmitis rates between 0.028% and 0.056%^(3^-^5)^. In contrast, research on the adverse effects of this procedure in Brazil is limited, and existing studies offer less data compared to international research^(6)^. The considerable variation in how intravitreal antiangiogenic therapy is administered-including factors such as the use of operating rooms versus office-based settings, eyelid specula, antiseptic preferences, and the choice of sterile scrubs and gloves-highlights the need for a deeper understanding of how these therapies are applied in everyday clinical practice^(7^,^8)^. Two studies are particularly noteworthy: Baudin et al., which found topical antibiotics ineffective in preventing endophthalmitis and suggested a potential risk when combining antibiotics with corticosteroids^(9)^, and Levinson et al., which observed a sevenfold reduction in endophthalmitis when povidone-iodine was used after eyelid speculum placement compared to other protocols^(10)^.

Several studies have shown that the efficacy of anti--VEGF treatments in real-world settings is lower than in randomized clinical trials, primarily due to factors such as patient demographics^(11)^, treatment adherence^(12^,^13)^, and injection frequency^(14^,^15)^. Like studies on adverse effects, real-world data on efficacy in Brazil is also limited.

This study aims to fill this gap by performing a thorough retrospective analysis of patient medical records from our ophthalmological center over the past decade. The goal was to gather data on intravitreal antiangiogenic injections, including the techniques used, medications administered, range of treated conditions, complication rates and types, and treatment efficacy. This study endeavor aims to improve therapeutic strategies and ultimately enhance the prognosis and quality of life for patients with retinal diseases.

## METHODS

This historical cohort study provides an in-depth review of medical records from anti-VEGF injections performed by a single surgeon between August 2011 and July 2023. The search for procedures was conducted using the following keywords in the electronic medical record system: intravitreal injection, Avastin, Lucentis, Eylia (the name of Eylea in Brazil), Vsiqq (the name of Beovu in Brazil), bevacizumab, ranibizumab, aflibercept, and brolucizumab. A CSV (comma-separated values) file was generated with data on all procedures performed. The file was analyzed using Microsoft^®^ Excel^®^ (Microsoft Corporation, Redmond, WA, USA), and patient names were extracted for further medical record review. Patient inclusion was not limited by diagnosis, reflecting the diversity of cases. No exclusion criteria were applied in this study. The primary aim was to assess the incidence of complications, including postinjection endophthalmitis, sterile inflammation, retinal detachment, and vitreous hemorrhage, in relation to variations in medication, injection techniques over time, and whether injections were performed bilaterally or unilaterally. We also conducted an efficacy analysis by comparing the best-corrected visual acuity (BCVA) before the first injection with the last recorded BCVA, as well as comparing the central subfield thickness (CST) on optical coherence tomography (OCT) from eyes that had more than 2 years of followup after the first injection. Only patients evaluated by authors were included, with BCVA assessed using the same standard Snellen chart and OCT exams performed on the same equipment (Spectralis OCT1, Heidelberg Engineering GmbH, Heidelberg, Germany). For the CST analysis, only patients diagnosed with AMD, diabetic macular edema (DME), and RVO-associated macular edema were included. A subgroup analysis was performed for the three most common diagnoses: DME, AMD, and RVO-associated macular edema, for both the CST and BCVA analyses.

Ethical approval for the study was granted by the Research Ethics Committee of the Hospital de Clínicas de Porto Alegre (Project number: 71178223.1.0000.5327). The study was exempt from obtaining informed consent.

### Intravitreal anti-VEGF injection method

All injections were performed in a surgical operating room or a designated sterile room specifically used for intravitreal injections. A lid speculum was used consistently for all patients, and injections were administered in the lower temporal quadrant, between 3 and 4 mm from the limbus. Preoperative topical anesthetic drops and topical povidone-iodine were routinely applied before the lid speculum placement. Starting in 2018, an additional drop of povidone-iodine was applied after inserting the lid speculum. Postinjection antibiotics were prescribed until 2017, after which they were discontinued. The injected volume was consistently 0.05 mL, delivered through a 30-gauge needle.

For bilateral injections, strict precautions were followed. After the first injection, all materials were replaced, fresh gloves were worn, and additional povidone-iodine was applied before administering the injection in the second eye.

### Treatment protocol

Patients with DME, AMD, and RVO-related macular edema were initially assigned to the pro re nata (PRN) treatment protocol before 2015 and to the treat-and--extend (T&E) protocol thereafter. Both protocols included loading injections during the first 3 months. In the PRN group, patients received monthly injections after the loading phase until OCT images showed no disease activity and BCVA was stable or improved compared to the last visit. Followup consisted of BCVA, dilated fundus examination, and OCT (one vertical and one horizontal scan through the fovea, as well as a macular cube) typically every 1-2 months, with no further injections unless disease activity recurred on OCT. In the T&E group, after the loading phase, followup visits could be extended if there was no disease activity on OCT and BCVA was stable or improved from the previous visit. The followup interval was extended by 4 weeks each time, starting from 4 weeks at baseline. If the disease-free interval reached 20 weeks, treatment was discontinued, with monthly or bimonthly monitoring using BCVA, dilated fundus examination, and OCT. If OCT revealed new disease activity, the followup and injection interval were reduced by 4 weeks, with a minimum of 4 weeks between visits. Patients with neovascular glaucoma or proliferative diabetic retinopathy (PDR) received injections as needed (e.g., significant increase in new vessels or vitreous hemorrhage). Patients with myopic neovascular membranes followed the PRN protocol.

All data were analyzed between November 2023 and August 2024 and recorded in a Microsoft^®^ Excel^®^ document (Microsoft Corporation, Redmond, WA, USA). Tables were also created for patients followed for more than 2 years, where BCVA and CST were recorded for subsequent statistical analysis. Statistical analysis was performed using SigmaPlot^®^ 15.0 (Grafiti LLC). Parametric data are presented as mean and standard deviation (SD). Non-parametric data are described using the median and interquartile range (IQR), and comparisons were made using the Wilcoxon signed-rank test. The chi-squared test was used to assess the relationship between categorical variables (complications associated with each medication).

## RESULTS

### Patient demographics

A total of 1,024 patients participated in the study, including 471 men (46%) and 553 women (54%), with a mean age of 65.9 years (SD, 9.7). In total, 1,310 eyes were treated during the study period.

### Injection profile

A total of 11,377 intravitreal injections were administered. Of these, 3,696 injections (32.48%) were part of bilateral procedures, while 7,681 (67.51%) were part of unilateral procedures. The breakdown of injection types was as follows: aflibercept (Eylea^®^ or Eylia^®^ in Brazil; Regeneron, Tarrytown, NY, USA), 6,833 (60.05%); ranibizumab (Lucentis^®^; Genentech/Roche, South San Francisco, CA, USA), 3,692 (32.45%); bevacizumab (Avastin^®^; Genentech, South San Francisco, CA, USA), 843 (7.40%); and brolucizumab (Beovu^®^ or Vsiqq^®^ in Brazil, Novartis, Basel, Switzerland), 9 (0.07%).

### Predominant diagnoses

The most common diagnoses were as follows: DME, 366 patients (35.74%); exudative AMD, 323 patients (31.54%); macular edema due to RVO, 187 patients (18.26%); PDR (with or without vitreous hemorrhage), 105 patients (10.25%); myopic neovascular membrane, 17 patients (1.66%); neovascular glaucoma, 16 patients (1.56%); macroaneurysm-associated macular edema, 4 patients (0.39%); neovascular membrane associated with angioid streaks, 2 patients (0.19%); neovascular membrane associated with chorioretinal scar, 2 patients (0.19%); Eales disease, 1 patient (0.09%); and hyperviscosity syndrome (multiple myeloma)-associated macular edema, 1 patient (0.09%).

The demographics are summarized in table 1.

**Table 1:** Demographics

	n (%)
Patient number	1,024 (100)
Eyes treated	1,310 (100)
Sex	
Female	553 (54.00)
Male	471 (46.00)
Injection number	11,377 (100)
Procedures	
Unilateral	7,681 (67.51)
Bilateral	3,696 (32.48)
Drug	
Aflibercept	6,833 (60.05)
Ranibizumab	3,692 (32.45)
Bevacizumab	843 (7.40)
Brolucizumab	9 (0.07)
Pathologies (patient number)	
DME	366 (35.74)
AMD	323 (31.54)
RVO-associated macular edema	187 (18.26)
PDR	105 (10.25)
Myopic neovascular membrane	17 (1.66)
Neovascular glaucoma	16 (1.56)
Other	10 (0.097)
Adverse effects	
Sterile vitritis	2 (0.00017)
Retinal detachment	1 (0.000087)
Infectious endophthalmitis	0 (0.00)

DME= diabetic macular edema; AMD= age-related macular degeneration; RVO= retinal vein occlusion; PDR= proliferative diabetic retinopathy.

### Safety profile

Mild adverse effects such as subconjunctival hemorrhage (hyposphagma) and ocular irritation/pain within the first two days postinjection were not included in the analysis. Notably, no cases of endophthalmitis were observed. Vitritis with decreased visual acuity was documented in two cases, both in male patients treated for DME and both following aflibercept injection (p<0.05 compared to other drugs, chi-squared test). One case occurred after a bilateral procedure and the other after a unilateral injection. Both cases improved with topical corticosteroid treatment, with no permanent visual loss. Additionally, one case of retinal detachment was diagnosed in a patient with a preexisting chorioretinal scar. This complication was successfully treated with vitrectomy and C3F8 gas.

### Medication trends over the years

**Figure 1:**
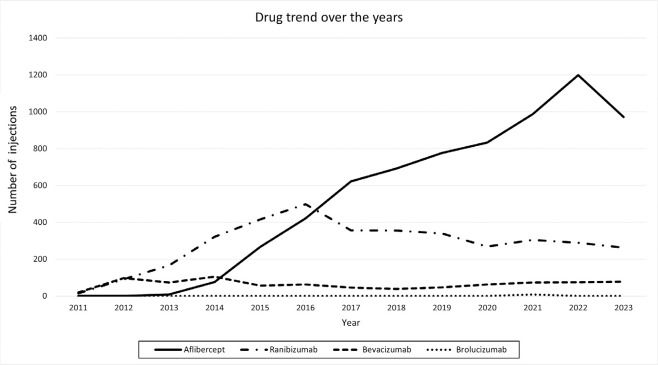
Medication trends over the years. Number of injections per year, categorized by drug. (Data for 2011 starts in August and data for 2023 ends in July)

A review of the injections from 2011 to 2023 showed a notable shift in the selection of anti-VEGF agents, as illustrated in figure 1. The data reveal a growing preference for aflibercept over time. While the use of ranibizumab increased initially, it began to decline from 2016 onward. Bevacizumab, though less commonly used overall, remained relatively stable throughout the study period.

### Efficacy outcomes

The median number of injections administered was 6 (IQR, 3-13). The patient with the highest number of injections underwent 136 procedures over an 8-year period, including both eyes.

**Figure 2:**
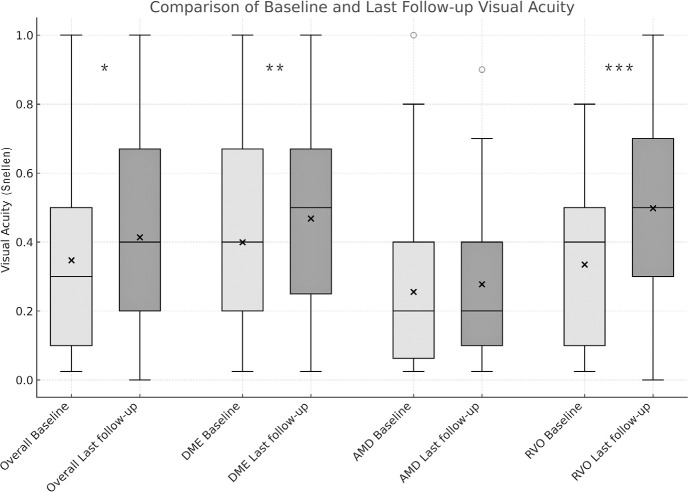
Best-corrected visual acuity: comparison of baseline and final followup

A total of 445 treated eyes from 328 patients completed more than 2 years of followup, with a median followup time of 4.05 years (IQR, 2.89-6.29). Among these long-term followup patients, 248 eyes had DME, 131 had AMD, 51 had RVO-related macular edema, 14 had DR, and 1 had neovascular glaucoma. The median BCVA before treatment was 0.3 (IQR, 0.1-0.5) (Snellen equivalent). The final BCVA measured a median of 0.4 (IQR, 0.2-0.67), representing a 5-letter gain on the ETDRS chart. This improvement in BCVA was statistically significant (p<0.001, Wilcoxon signed-rank test). When stratifying by disease type and evaluating the three most common conditions in the sample-DME, exudative AMD, and RVO-related macular edema-we found that, for DME patients, the median BCVA improved from 0.4 (IQR, 0.2-0.67) to 0.5 (IQR, 0.25-0.67), with a statistically significant difference (Wilcoxon signed-rank test, p=0.007). For patients with exudative AMD, the median BCVA before treatment was 0.2 (IQR, 0.0625-0.4), and after treatment, it was 0.3 (IQR, 0.1-0.4), with no statistically significant difference (Wilcoxon signed-rank test, p=0.217). For patients with RVO-related macular edema, the median BCVA improved from 0.4 (IQR, 0.1-0.5) to 0.5 (IQR, 0.3-0.7), with a statistically significant difference (Wilcoxon signed-rank test, p=0.017). The baseline and final BCVA differences are presented in figure 2.

Boxplots for BCVA showing median, IQR, and 1.5 x IQR (whiskers) for eyes followed for more than 2 years, overall and in subgroups: diabetic macular edema (DME), exudative age-related macular degeneration (AMD), and macular edema related to retinal vein occlusion (RVO). Overall data includes all eyes followed for more than 2 years. Baseline is shown in light gray and final followup in dark gray. X represents the mean. Circles denote outliers. *p<0.001, Wilcoxon signed-rank test. ** p=0.007, Wilcoxon signed-rank test. ***p=0.017, Wilcoxon signed-rank test

**Figure 3:**
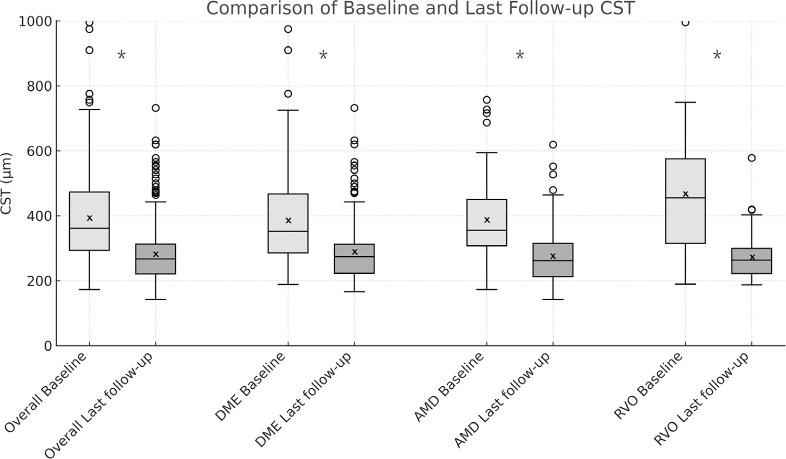
Central subfield thickness: comparison of baseline and final followup

Regarding anatomical outcomes, for eyes with diseases eligible for CST analysis (DME, AMD, and RVO-related macular edema), the median CST before treatment was 361.00 microns (IQR, 293-473), and the final measured CST was 267.00 microns (IQR, 220.75-312.25). This decrease in CST was statistically significant (p<0.001, Wilcoxon signed-rank test). When analyzing by disease type, eyes with DME had a median pre-treatment CST of 352.00 microns (IQR, 285.50-467.50) and a posttreat-ment CST of 269.50 microns (IQR, 231.50-327.50), with a statistically significant difference (p<0.001, Wilcoxon signed-rank test). Eyes with exudative AMD had a median pre-treatment CST of 355.50 microns (IQR, 307.25-450.00) and a posttreatment CST of 267.00 microns (IQR, 225-315), showing a significant difference (p<0.001, Wilcoxon signed-rank test). Eyes with RVO-related macular edema had a median pre--treatment CST of 455 microns (IQR, 315-575) and a posttreatment CST of 276.50 microns (IQR, 220-332), also demonstrating a significant difference (p<0.001, Wilcoxon signed-rank test). The CST results are presented in figure 3.

Boxplots for CST showing median, IQR, and 1.5 x IQR (whiskers) from eyes followed for more than 2 years, overall and in subgroups: DME, exudative AMD, macular edema related to RVO. Overall data includes these three groups and excludes other pathologies. Baseline is in light gray and final followup is in dark gray. X represents the mean. Circles are outliers. *p < 0.001, Wilcoxon signed-rank test

## DISCUSSION

The introduction of intravitreal injections, particularly antiangiogenic agents, has significantly advanced ophthalmology. Prior to these treatments, conditions like exudative AMD, DME, and RVO-related macular edema presented significant challenges, often leading to severe visual impairment due to the lack of effective treatments. These injections have transformed treatment, providing meaningful visual improvements for many patients.

Our findings align with broader scientific literature, demonstrating that these injections are both effective and safe when administered properly. A key observation from our data is the absence of endophthalmitis, a serious complication, emphasizing the importance of strict aseptic techniques during administration. The use of povidone-iodine is crucial in preventing endophthalmitis, and the safety of performing bilateral injections on the same day is supported, as no increased complications were observed compared to unilateral procedures.

An interesting finding in our study, consistent with previous research, is that sterile vitritis occurs more frequently with aflibercept compared to other medications, though it did not result in irreversible vision loss in this study.

The most severe adverse event was a single case of retinal detachment among 11,377 injections, which is within the expected range for this procedure. In the study by Storey et al., one retinal detachment occurred for every 7,692 injections, with vitrectomy being the preferred treatment^(16)^.

The data also suggest a shift in the preference for anti-VEGF agents over time in our cohort, with a noticeable trend toward using aflibercept over ranibizumab and bevacizumab in managing various retinal diseases. This shift may stem from the belief among clinicians and patients that aflibercept offers superior efficacy compared to ranibizumab. However, a thorough review of the literature indicates that the clinical outcomes between these two anti-VEGF agents show minimal, if any, difference, with some studies reporting better results in patients with the poorest BCVA^(17)^ and a greater reduction in CST, even though long-term BCVA outcomes are similar^(18^,^19)^. It is important to note that the limited use of bevacizumab in this study is due to it being off--la-bel and not reimbursed by insurance companies in Brazil, despite being a more affordable option with efficacy comparable to ranibizumab^(20)^. The efficacy analysis we performed, acknowledging the limitations of retrospective, uncontrolled studies, shows satisfactory results consistent with similar global studies. We observed an improvement in visual acuity across the entire patient group, with further improvement in patients with DME and RVO-related macular edema, and stability in visual acuity for patients with exudative AMD. Ciulla et al. reported a 10-letter improvement by the end of the first year in patients treated for DME^(15)^, while Payne et al. observed an improvement of 2-9 letters by the second year, along with a significant reduction in CST, from approximately 430 to 340 microns^(21)^. Additionally, a real-world study on AMD by Hujanen et al. found stable BCVA at the end of the fourth year, aligning closely with our results^(22)^, while Yang et al.’s study showed BCVA improvements after 48 months of followup^(23)^. Although these functional results are lower than those from clinical trials^(24^-^26)^, as seen in other real-world comparisons, it is important to emphasize that these are serious diseases leading to significant and often irreversible visual impairment, which can be mitigated through antiangiogenic treatment, even in developing countries. Our study supports the global findings on safety and efficacy and highlights the subtle differences between agents, particularly aflibercept. This study has several limitations that should be emphasized: it is retrospective, may have potential followup biases, was conducted at a single center with all procedures performed by the same ophthalmologist, and involved a broad range of diseases. Additionally, confounding factors related to visual acuity, such as cataracts and cataract surgery, as well as factors affecting CST, such as macular ischemia, must be considered. It is challenging to assess the extent to which loss to followup may have impacted the efficacy results. On one hand, patients may not return due to improved visual acuity, or they may discontinue treatment if they did perceive no improvement. Future studies with a larger number of procedures are needed to confirm these findings, which may become easier with the increased use of digital medical record systems. Despite these limitations, this study represents the largest survey conducted in Brazil on the safety and efficacy of intravitreal anti-VEGF injections for retinal diseases.
